# *Mycobacterium tuberculosis*–derived circulating cell-free DNA in patients with pulmonary tuberculosis and persons with latent tuberculosis infection

**DOI:** 10.1371/journal.pone.0253879

**Published:** 2021-06-24

**Authors:** Sheng-Wei Pan, Wei-Juin Su, Yu-Jiun Chan, Fan-Yi Chuang, Jia-Yih Feng, Yuh-Min Chen

**Affiliations:** 1 Department of Chest Medicine, Taipei Veterans General Hospital, Taipei, Taiwan; 2 School of Medicine, National Yang Ming Chiao Tung University, Taipei, Taiwan; 3 Institute of Public Health, National Yang Ming Chiao Tung University, Taipei, Taiwan; 4 Division of Infectious Diseases, Department of Medicine, Taipei Veterans General Hospital, Taipei, Taiwan; 5 Division of Microbiology, Department of Pathology and Laboratory Medicine, Taipei Veterans General Hospital, Taipei, Taiwan; The University of Georgia, UNITED STATES

## Abstract

**Objectives:**

The timely diagnosis of pulmonary tuberculosis (PTB) is challenging. Although pathogen-derived circulating cell-free DNA (cfDNA) has been detected in humans, the significance of *Mycobacterium tuberculosis* (MTB)-cfDNA detection in patients with PTB remains unclear.

**Methods:**

This study enrolled patients with PTB and persons with latent tuberculosis infection (LTBI) as the study and control groups, respectively, from 2018 to 2020. We measured interferon-γ levels and calculated blood monocyte-to-lymphocyte ratio (MLR). We conducted plasma cfDNA extraction, quantitative polymerase chain reaction (qPCR), and droplet digital PCR targeting the *IS6110* gene of MTB. We calculated the sensitivity and specificity of using MTB-cfDNA to identify PTB and analyzed the factors associated with PTB diagnosis and MTB-cfDNA positivity.

**Results:**

We enrolled 24 patients with PTB and 57 LTBI controls. The sensitivity of using MTB-cfDNA to identify PTB was 54.2%(13/24) in total and 46.2%(6/13) in smear-negative cases. Two LTBI controls (3.5%) tested positive for MTB-cfDNA, indicating a specificity of 96.5%(55/57). By using MTB-cfDNA positivity and an MLR ≥0.42 to identify PTB, sensitivity increased to 79.2%(19/24). Among patients with PTB, MTB-specific interferon-γ levels were higher in MTB-cfDNA positive participants than in those who tested negative (7.0 ±2.7 vs 2.7±3.0 IU/mL, *p*<0.001). MTB-cfDNA levels declined after 2 months of anti-tuberculosis therapy (*p*<0.001).

**Conclusion:**

The sensitivity of using MTB-cfDNA to identify PTB in participants was 54.2%, which increased to 79.2% after incorporating an MLR ≥0.42 into the analysis. MTB-cfDNA positivity was associated with MTB-specific immune response, and MTB-cfDNA levels declined after treatment. The clinical value of MTB-cfDNA in PTB management necessitates further investigation.

## Introduction

Pulmonary tuberculosis (PTB) is caused by the *Mycobacterium tuberculosis* (MTB) complex, and bacteriological evidence is generally required to confirm diagnosis [1]. Timely diagnosis and rapid treatment of PTB improves clinical outcomes and reduces the risk of transmission [2]. However, the time required to diagnose PTB (3–8 weeks) may be prolonged in patients with subclinical infection and those with a negative sputum-smear test for acid-fast bacilli [3, 4]. For early diagnosis, invasive procedures or alternative tests may be required. Some candidate biomarkers are used for rapid diagnosis; however, the interferon-γ release assays (IGRA) for the diagnosis of latent TB infection (LTBI) cannot distinguish between persons with LTBI and patients with PTB [5]. Recently, blood monocyte-to-lymphocyte ratio (MLR) has exhibited potential for differentiating patients with active PTB from those with LTBI; however, the discriminative value for adults in TB endemic regions is uncertain [6]. Therefore, developing noninvasive screening tests for the diagnosis of PTB remains worthwhile [1].

Detecting pathogen-derived cell-free DNA (cfDNA) in human blood, namely circulating cfDNA, has been used to identify causative etiology in patients with infectious diseases [7]. Broken microbial gene fragments derived from pathogens or dying human cells/tissues are believed to be released into the acellular fraction of blood [8]. In 2016, Ushio et al. reported that MTB-specific insertion sequence 6110 (*IS6110*)-cfDNA can be detected in the plasma of patients with PTB by using droplet digital polymerase chain reaction (ddPCR) [9]. Subsequently, Click et al. used quantitative PCR (qPCR) methods to demonstrate that MTB-cfDNA could be detected in the plasma of nearly half of patients with PTB, even in the absence of MTB bacteremia [10]. However, the sensitivity of using MTB-cfDNA to identify PTB remains too low for clinical application. In addition, whether the presence of blood MTB-cfDNA is specific for patients with active PTB but not persons with LTBI is unknown. Because proof-of-principle studies were conducted on patients with smear-positive PTB and controls without LTBI, the clinical significance of using MTB-cfDNA to identify PTB remains unclear [11]. Furthermore, although one study reported that certain characteristics of patients with PTB may be associated with MTB-cfDNA detectability [9], none have investigated the immunologic determinants of positive results and the effect of antituberculosis therapy on MTB-cfDNA positivity.

By enrolling patients with LTBI but without PTB as the controls, this study was able to investigate the value of using MTB-cfDNA to diagnose PTB and compare its discriminative ability with MLR. We also evaluated factors associated with MTB-cfDNA positivity and assessed changes in MTB-cfDNA levels after treatment.

## Methods

### Study design and enrollment

We conducted this prospective study at Taipei Veterans General Hospital in Taiwan. We included adult patients from outpatient clinics with active PTB who had (1) respiratory samples that were culture-positive for MTB or (2) a biopsy indicating active lung lesions with compatible pathologic results and with tissue that tested positive for the *IS6110* gene from June 2018 to May 2019. We also enrolled control persons with a recent history of TB contact (exposure to a patient with PTB within 6 months) and LTBI from October 2018 to September 2020. In Taiwan, since 2016, persons with a history of TB contact and with LTBI have received chest radiographic examinations to exclude the possibility of PTB and have received prophylactic therapy for LTBI. The enrollment criteria for LTBI controls were having reported household or equivalent close contact with patients with PTB within 6 months but without an abnormal chest radiograph indicating active PTB or a history of MTB infection and testing positive for IGRA [6].

The exclusion criteria were (1) a history of MTB infection and (2) current extrapulmonary TB or human immunodeficiency virus infection. All participants provided written informed consent approved by the Institutional Review Board of Taipei Veterans General Hospital (Nos. 2017-12-001C, 2018-10-017A, and 2019-07-003C).

### Blood sampling schedule and process

At baseline, we collected peripheral blood samples from patients with PTB and controls with LTBI before treatment. For patients with PTB, we performed blood tests again at 2 months after anti-TB therapy. We used K2-EDTA vacutainer tubes (BD) to collect blood (8–10 mL) for plasma preparation. Within 2 h, we obtained plasma samples by centrifuging the blood for 10 min at 1500 rpm (430 g) [9]. We extracted cfDNA from 400 μL of each plasma sample by using the QIAamp Blood DNA Mini Kit (250, Qiagen) with a final elution volume of 50 μL [12]. We measured the concentration of cfDNA samples through the Nanodrop method. We stored the cfDNA samples in microcentrifuge tubes at −80°C for subsequent PCR testing in batches.

### Real-time qPCR and ddPCR

Using the plasma cfDNA samples, we performed qPCR to detect the target MTB-specific cfDNA, namely *IS6110* (GenBank accession No X17348.1). We used the primer and probe set developed by Ushio et al. to amplify a 71-bp region of the MTB-specific *IS6110* gene [9]. Specifically, the primers we used for *IS6110* amplification were *IS6110* forward (5’-GGCGTACTCGACCTGAAAGA-3’) and *IS6110* reverse (5’-CTGAACCGGATCGATGTGTA-3’). The internal probe we used was *IS6110* probe (5’-[FAM]-CCACCATACGGATAGGGGAT-[BHQ-1]-3’) that was labeled by a reporter dye (6-carboxyfluorescein, FAM) on the 5’ end of the probe and a quencher dye (Black Hole Quencher, BHQ-1) on its 3’ end. The reaction mixture (20 μL in total) consisted of (1) 2× ChamQ Universal U+ Probe Master Mix (Vazyme #Q713), (2) 300 nM primers and 300 nM probes, and (3) 3 μL of cfDNA samples. The Applied Biosystems 7500 Fast Real-Time PCR System was used to set the PCR conditions for initial incubation to 37°C for 2 min, 95°C for 1 min, and 45 cycles at 95°C for 10 s and at 60°C for 30 s. We tested samples in duplicate, and if the difference between the paired cycle threshold (Ct) value of one sample was >1, we performed qPCR for the sample again in triplicate. We recorded the Ct value as 45 if the value was >45. We used positive controls (DNA extraction of MTB H37Rv) and no-template controls (NTCs) for each qPCR assay.

We performed ddPCR to validate the qPCR findings of all plasma samples from patients with PTB and in samples with Ct values of <45 from LTBI controls in the manner described by Ushio et al. and in accordance with the Minimum Information for Publication of Quantitative Digital PCR Experiments (digital MIQE) guidelines [9, 13]. The ddPCR reaction mixture included 10 μL of ddPCR Supermix for the probes (BioRad), 900 nM primers, 250 nM probes, and 5 μL of cfDNA samples. We added ultrapure DNase- and RNase-free water to the reaction mixture, which resulted in a final volume of 20 μL. We also used positive controls and NTCs to rule out a false positive result. We used the QX200 Droplet Generator (BioRad) for microdroplet generation, the T100 Thermal Cycler (BioRad) for *IS6110* amplification, and the QX200 Droplet Reader (BioRad) to measure the fluorescence intensity signal of each droplet in the emulsion. We applied Poisson distribution to the exported fluorescence signal data to estimate the copy numbers [9]. We conducted the ddPCR experiments at the Center of Genomic Medicine at National Taiwan University.

### Data collection and other measurements

We collected clinical data comprising comorbidities and radiographic findings. For patients with PTB, we obtained the results of sputum acid-fast bacilli smears and cultures and of lung biopsies. Using the QuantiFERON-TB Gold In-Tube test, we performed IGRA on patients with PTB and on persons with a history of TB contact and LTBI upon enrollment [14]. We recorded the levels of interferon-γ (IFN-γ, IU/mL) in test tubes without stimulation (nil tube) and with stimulation by using MTB-specific antigen (TB-Ag) and mitogen. A positive IGRA result indicating LTBI was defined as a difference in IFN-γ levels between the TB-Ag and nil tubes of >0.35 IU/mL and >25% of the nil value [15]. We recorded the results of routine white blood cell count and differential count (including monocytes and lymphocytes) and also calculated MLR at baseline. We observed LTBI controls since enrollment to February 2021 to ensure no TB development.

### Statistical analysis

We presented data as a number (%) and a mean ± SD or median with interquartile range (IQR) as appropriate. We performed Student’s *t* test or the Mann–Whitney *U* test to compare continuous variables between groups. To determine the optimal cutoff for Ct values, we performed receiver operating characteristic (ROC) curve analyses. We calculated the sensitivities and specificities for detecting MTB-cfDNA to diagnose PTB. We used a logistic regression to identify factors associated with PTB and MTB-cfDNA positivity, respectively. We included variables with a univariate *p* value of <0.05 in the multivariate analysis. Finally, we used a paired *t* test to compare the Ct values of *IS6110*-qPCR before and after 2 months of treatment in the patients with PTB. We used SPSS 18 (SPSS Inc.) for all statistical analyses.

## Results

### Characteristics of participants

**Fig 1:**
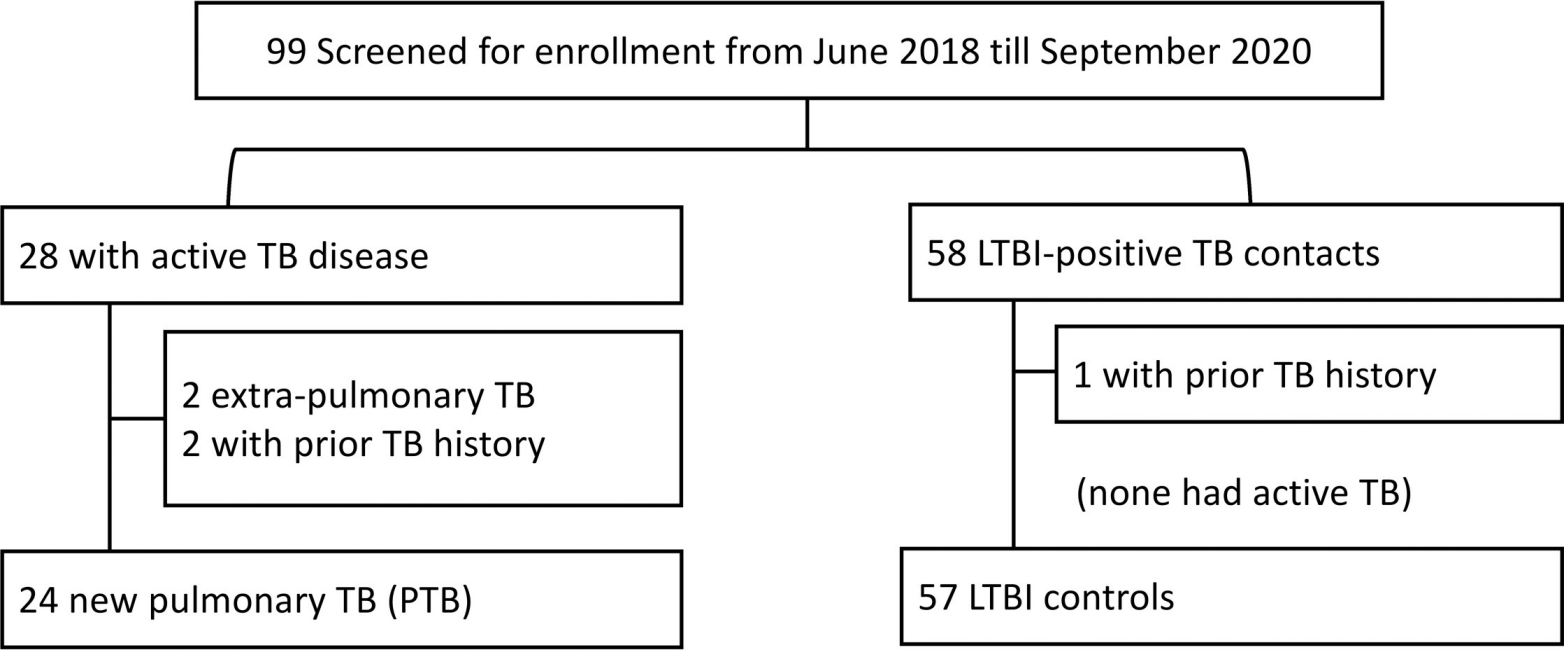
Flowchart of participant enrollment. Abbreviations: LTBI, latent tuberculosis (TB) infection.

**Fig 2:**
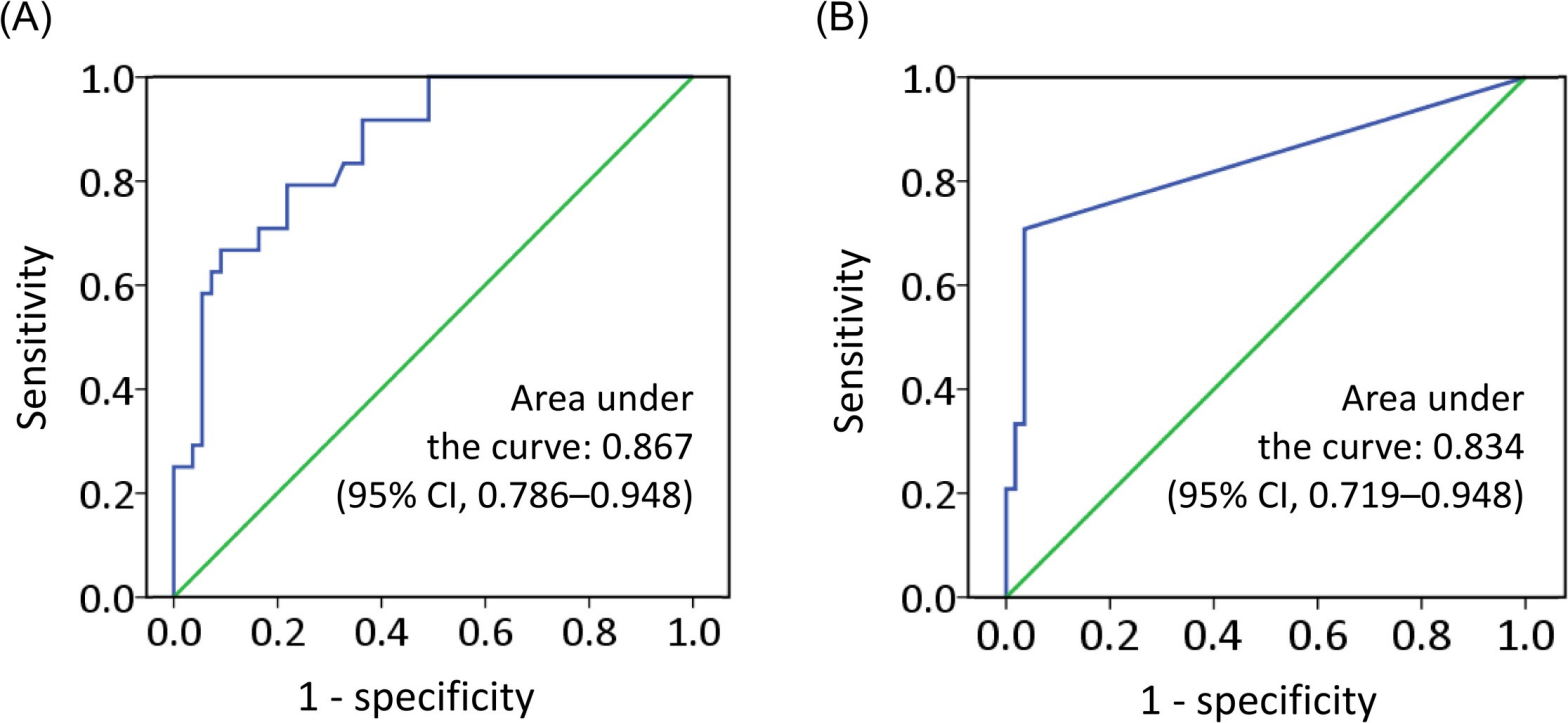
ROC curve analysis of (A) blood MLR and (B) Ct value of qPCR targeting MTB-specific *IS6110* in plasma cell-free DNA (MTB-cfDNA) to identify patients with PTB.

We enrolled 24 patients with PTB and 57 persons with a history of TB contact and with LTBI (Fig 1). As presented in Table 1, the characteristics of the patients with PTB and the LTBI controls differed in gender ratio, body mass index, smoking status, IGRA responses, and lymphocyte and monocyte counts. In particular, the MLR in the PTB group was higher than that in the control group (0.52 ± 0.22 vs 0.28 ± 0.10, *p* < 0.001). ROC curve analysis revealed that the optimal MLR cutoff for identifying PTB in participants was 0.42 (Fig 2A).

**Table 1:** Characteristics of patients with PTB and controls with LTBI (*n* = 81).

Variable	PTB cases (n = 24)	LTBI controls (n = 57)	P value
Age, years	62.1±21.0	63.7±17.4	0.727
Male sex	17 (71)	30 (43)	0.028
BMI (kg/m2)	21.0±3.4	23.4±3.2	0.003
Ever smoker	15 (63)	16 (23)	0.003
Diabetes	6 (25)	12 (17)	0.680
Malignancy	4 (17)	4 (6)	0.187
CKD	1 (4)	2 (3)	1.000
BCG scar	18 (78)	53 (79)	1.000
IFN-γ (IU/mL)			
MTB-Ag–nil	5.30±3.73	2.78±2.91	0.005
Mitogen–nil	7.63±3.04	9.35±1.37	0.005
Differential counts^a^			
Lymphocyte (k/mm^3^)	1.37±0.42	1.85±0.53	<0.001
Monocyte (k/mm^3^)	0.68±0.36	0.51±0.21	0.006
MLR	0.52±0.22	0.28±0.10	<0.001
qPCR Ct value	38.06 [35.73–45]	45.0 [45.0–45.0]	<0.001

^a^For two patients with LTBI, no data on blood differential count or MLR were available.

Abbreviations: BCG, Bacillus Calmette–Guérin vaccine; BMI, body mass index; CKD, chronic kidney disease; IFN-γ, interferon-gamma; MLR, monocyte to lymphocyte ratio; MTB-Ag, *Mycobacterium tuberculosis*–specific antigen stimulation; qPCR Ct value, quantitative polymerase chain reaction–derived cycle threshold value.

### Results of *IS6110*-targeted qPCR and ddPCR

**Fig 3:**
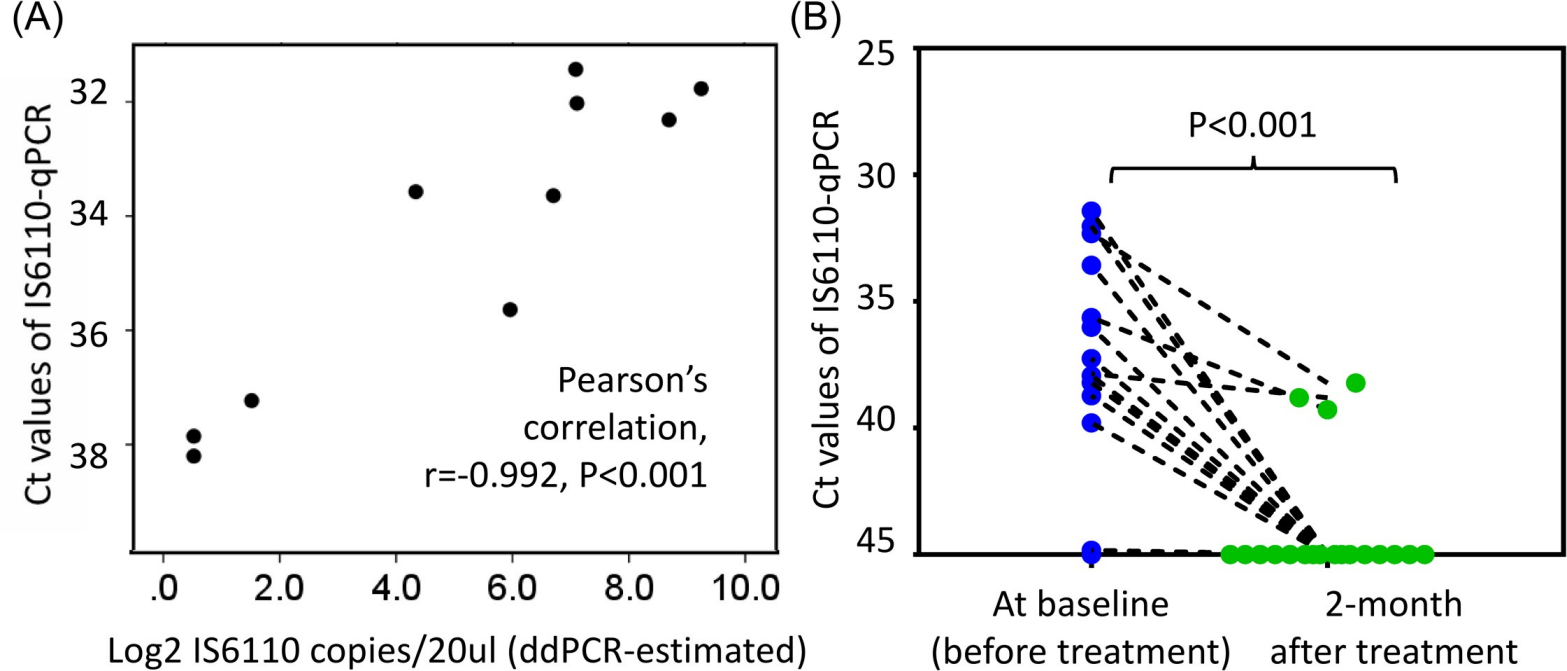
(A) Correlation between *IS6110*-qPCR Ct values and ddPCR-estimated copy number (Log2) and (B) posttherapy changes in Ct values of *IS6110*-qPCR in 10 patients with MTB-cfDNA-positive PTB.

The *IS6110*-qPCR results revealed that the median Ct values were 38.06 and 45.0 in the PTB and LTBI groups, respectively (IQRs: 35.73–45 and 45–45, respectively; *p* < 0.001). In the ROC curve analysis, the calculated area under the ROC curve for *IS6110*-qPCR in the detection of PTB was 0.834 (95% CI, 0.719–0.948, Fig 2B), and the optimal cutoff Ct value was 45.0. However, as presented in Table 2, *IS6110*-targeted ddPCR revealed no positive result in samples with a Ct value >38.2. The correlation analysis indicated that qPCR-derived Ct values were significantly correlated with ddPCR-estimated *IS6110* copy numbers/20 μL (converted to Log2; Pearson’s correlation *r* = −0.992, *p* < 0.001, *n* = 10; Fig 3A). By using the modified cutoff Ct value of 38.2 to define MTB-cfDNA positivity, the qPCR assay identified MTB-cfDNA in 13/24 (54.2%) plasma samples from patients with PTB and 2/57 (3.5%) samples from LTBI controls, yielding a sensitivity and specificity of 54.2% and 96.5%, respectively. For the 13 patients with PTB with smear-negative sputum, the sensitivity of using MTB-cfDNA positivity to identify PTB was 46.2% (6/13).

**Table 2:** Results of *IS6110*-targeted PCR (*n* = 81).

Participants	IS6110-target qPCR Ct values	ddPCR-estimated IS6110 copy/20ul	MTB-specific IFN-γ (IU/mL)
PTB cases			
1	31.43	136.0	6.43
2	31.77	612.0	8.12
3	32.02	138.0	9.90
4	32.31	416.0	9.94
5	33.57	20.0	1.46
6	35.64	62.0	6.99
7	36.01	ND	7.74
8	36.31	ND	9.93
9	37.23	2.8	9.95
10	37.25	0	6.17
11	37.85	1.4	4.79
12	37.93	0	9.19
13	38.20	1.4	3.58
14	38.58	0	0.84
15	38.73	0	0.48
16	39.80	0	0.10
17	44.83	0	0
18–24	>45.00	0	4.50±3.65
LTBI controls			
1	33.64	104.0	7.60
2	36.93	0	3.14
3–57	>45.00	-	2.69±2.88

Abbreviations: ddPCR, digital droplet polymerase chain reaction (PCR); LTBI, latent tuberculosis infection; ND, “not done” because of inadequate samples for ddPCR tests; MTB-specific IFN-γ, interferon-gamma released after *Mycobacterium tuberculosis*–specific antigen stimulation; PTB, pulmonary tuberculosis (TB); qPCR Ct value, quantitative PCR–derived cycle threshold value.

### MTB-cfDNA plus MLR to identify PTB

In the multivariate analysis (Table 3), independent factors for discriminating PTB from LTBI included an MLR >0.42 and MTB-cfDNA positivity after adjustment for cofactors. As displayed in Table 4, we compared the accuracy of using MTB-cfDNA and an MLR >0.42 to identify PTB. The sensitivity of MTB-cfDNA positivity was lower than that of MLR >0.42 (54.2% [95% CI, 34.2%–74.1%] vs 66.7% [47.8%–85.5%]) although its specificity was slightly higher (96.5% [34.2%–74.1%] vs 90.9% [83.3%–98.5%]). After using MTB-cfDNA positivity or an MLR >0.42 to detect PTB, sensitivity increased to 79.2% (19/24) with a moderately reduced specificity of 87.3% (48/55). For 13 smear-negative patients with PTB, the sensitivity for PTB diagnosis also increased to 69.2% (9/13).

**Table 3:** Analysis of independent factors for discriminating patients with PTB and controls with LTBI.

Variable	Crude OR (95% CI)	P value	Adjusted OR (95% CI)	P value
Age, years	0.995 (0.970–1.022)	0.723	0.812 (0.955–1.037)	0.812
Male sex	3.339 (1.198–9.310)	0.021	13.613 (6.000–309.06)	0.101
BMI (kg/m2)	0.785 (0.658–0.936)	0.007	0.561 (0.353–0.892)	0.015
Ever smoker	4.667 (1.691–12.879)	0.003	0.899 (0.100–8.103)	0.924
Diabetes	1.214 (0.427–3.454)	0.716		
Malignancy	3.600 (0.740–17.520)	0.113		
CKD	1.196 (0.103–13.846)	0.886		
BCG scar	1.114 (0.346–3.590)	0.856		
IFN-γ (IU/mL)				
MTB-Ag—nil	1.248 (1.007–1.445)	0.003	1.115 (0.836–1.488)	0.458
MLR >0.42^a^	20.00 (5.723–69.888)	<0.001	9.917 (1.825–53.873)	0.008
MTB-cfDNA positivity^b^	32.50 (6.410–164.778)	<0.001	54.447 (2.667–1111.5)	0.009

^a^For two patients with LTBI, no data on MLR were available.

^b^Positivity was defined as having an *IS6110*-target qPCR Ct value of ≤38.2.

Abbreviations: BCG, Bacillus Calmette–Guérin vaccine; BMI, body mass index; cfDNA, cell-free DNA; CKD, chronic kidney disease; IFN-γ, interferon-gamma; MLR, monocyte to lymphocyte ratio; MTB-Ag, *Mycobacterium tuberculosis*–specific antigen stimulation.

**Table 4:** Accuracy of MTB-cfDNA positivity and an MLR >0.42 for detecting PTB.

Biomarkers	Sensitivity, (n/N)	Specificity, (n/N)	Positive predictive value, (n/N)	Negative predictive value, (n/N)
MLR >0.42^a^	66.7% (16/24)	90.9% (50/55)	76.2% (16/21)	86.2% (50/58)
MTB-cfDNA^b^	54.2% (13/24)	96.5% (55/57)	86.7% (13/15)	83.3% (55/66)
Any-positive	79.2% (19/24)	87.3% (48/55)	73.1% (19/26)	90.6% (48/53)

^a^For two patients with LTBI, no data on MLR were available, and both tested negative for MTB-cfDNA.

^b^Positivity was defined as having an *IS6110*-target qPCR Ct value of ≤38.2.

Abbreviations: MTB-cfDNA, *Mycobacterium tuberculosis*–derived cell free DNA.

### Factors associated with MTB-cfDNA positivity in patients with PTB

As presented in Table 5, MTB-cfDNA–positive patients with PTB had increased levels of MTB-specific IFN-γ responses (7.25 ± 2.71 vs 2.99 ± 3.52 IU/mL, *p* = 0.003) when compared with those who tested negative. No statistical relationship between MTB-cfDNA positivity and sputum-smear positivity was identified. In addition, no significant difference was observed in the Ct values between patients with different sputum-smear grades (see S1 File). MTB-specific IFN-γ responses were significantly associated with MTB-cfDNA positivity after adjustment (*p* = 0.027). Among the 13 PTB patients with MTB-cfDNA positivity, 10 provided plasma samples after 2 months of treatment. With a cutoff Ct value of 38.2, none of the patients tested positive for MTB-cfDNA through qPCR assay. The median Ct value for the *IS6110*-targeted qPCR assay increased from 35.83 (IQR: 32.2–37.9) at baseline to 45.0 (39.2–45.0) after 2 months of anti-TB therapy (*p* < 0.001, paired *t* test, *n* = 10; Fig 3B).

**Table 5:** Characteristics of patients with PTB stratified by MTB-cfDNA positivity and analysis of factors associated with MTB-cfDNA positivity.

Variable	MTB-cfDNA positive		P value	Multivariate analysis	P value
	Yes (n = 13)	No (n = 11)		Adjusted OR (95% CI)	
Age, years	66.4±19.9	57.0±22.1	0.284	1.073 (0.990–1.163)	0.085
Male sex	9 (69)	8 (73)	1.000	0.690 (0.060–7.959)	0.766
BMI (kg/m2)	21.9±3.9	20.0±2.4	0.173		
Ever smoker	8 (62)	7 (64)	1.000		
Diabetes	5 (38)	1 (9)	0.166		
Malignancy	2 (15)	2 (18)	1.000		
CKD	1 (8)	0	1.000		
Fever	2 (15)	0	0.482		
BCG scar	9 (69)	9 (90)	0.339		
Smear positive	7 (54)	4 (36)	0.444		
Multi-lobar or cavitary lesion	9 (69)	5 (45)	0.408		
IGRA positive	13 (100)	9 (82)	0.199		
IFN-γ (IU/mL)					
MTB-Ag—nil	7.25±2.71	2.99±3.52	0.003	1.772 (1.117–2.809)	0.015
MLR >0.42	10 (77)	6 (55)	0.390		

Abbreviations: BCG, Bacillus Calmette–Guérin vaccine; BMI, body mass index; cfDNA, cell-free DNA; CKD, chronic kidney disease; IFN-γ, interferon-gamma; IGRA, interferon-gamma release assay; MLR, monocyte to lymphocyte ratio; MTB-Ag, *Mycobacterium tuberculosis* (MTB)-specific antigen stimulation.

## Discussion

This prospective study verified that circulating MTB-cfDNA was detectable in 54.2% of patients with PTB with a specificity of 94.3%. By using MTB-cfDNA positivity or an MLR >0.42 to detect PTB, sensitivity increased to 79.2%, with a specificity of 87.3%. Notably, the sensitivity for PTB detection in smear-negative patients increased from 46.2% to 69.2%. Moreover, we observed that MTB-cfDNA positivity in patients with PTB was correlated with MTB-specific IFN-γ responses and that MTB-cfDNA levels decreased after anti-TB treatment. Furthermore, this study provided the first microbiologic evidence of MTB infection, namely MTB-cfDNA, in persons with LTBI but without PTB. Accordingly, the findings suggest that MTB-cfDNA is an immune-associated microbial biomarker for the detection and monitoring of MTB infection.

To develop rapid, noninvasive tests for identifying PTB, studies have evaluated the feasibility of detecting MTB-derived genes in blood or in plasma/serum [8, 16]. Critical to the line of research, by using ddPCR to target a 71-bp *IS6110* gene fragment, Ushio et al. detected MTB-cfDNA in patients and reported a sensitivity of 65% and a specificity of 93% for PTB detection [9]. Although it may provide more precise quantification of cfDNA than qPCR, ddPCR is three times as expensive and therefore may be not suitable as a standard diagnostic test [17]. In a related study, Click et al. used qPCR targeting another 106-bp *IS6110* gene fragment for PTB detection, which demonstrated a sensitivity of 45% [10]. However, the cutoff qPCR-Ct value remained unconfirmed without the inclusion of control participants and validation by ddPCR. In addition, both studies restricted their study participants to smear-positive patients, in whom timely diagnosis by using conventional methods is naturally feasible. By contrast, our study verified that circulating MTB-cfDNA was detectable with the qPCR method in half of the patients with PTB, including those with smear-negative tests (46.2%, 6/13). For the smear-negative group, our finding is consistent with a study reporting that an *IS6110*-ddPCR assay detected PTB in 47.4% (9/19) of smear-negative patients [11].

Nevertheless, others’ findings and ours suggest that MTB-cfDNA alone is not sufficiently sensitive for detecting PTB in clinical practice. In addition to MTB-cfDNA, the results of the multivariate analysis in our study indicated that MLR was another independent factor for discriminating PTB from LTBI. A cohort study in Italy reported that an MLR of >0.30 had a sensitivity and specificity of 85.1% and 85.7%, respectively, in detecting PTB [6]. However, our study indicated that an MLR of >0.42 had a relatively low sensitivity (66.7%) for detecting PTB. Notably, the use of either an MRL >0.42 or MTB-cfDNA positivity resulted in 79.2% sensitivity and 87.3% specificity for PTB detection. Because this change was also true for the smear-negative subgroup, we suggest that integrating MTB-cfDNA and other biomarkers into PTB detection should be considered a potential method for timely PTB detection in patients suspected of having TB.

To ensure the reliability of MTB-cfDNA for PTB diagnosis, we included those with a history of TB contact and with LTBI but without active PTB as the controls. We also used ddPCR to confirm the appropriate qPCR Ct values for obtaining positive results. According to the qPCR results, two TB contacts with LTBI tested positive for *IS6110*-cfDNA. Because one of these participants also tested positive according to the ddPCR assay and both of them had significantly elevated IGRA responses, we believe that they both had MTB infection with circulating MTB-cfDNA. We also believe that the two patients with LTBI may have been in the incipient TB stage at the time of enrollment; however, they did not develop active TB during follow-up because they received prophylactic therapy for LTBI [18]. This suspicion may be corroborated by our observation that MTB-cfDNA levels declined after anti-TB therapy, suggesting that a smaller release of MTB-cfDNA occurred after MTB treatment and clearance. To our knowledge, our study is the first to report that certain persons with LTBI tested positive for MTB-cfDNA, which suggests that plasma MTB-cfDNA may be a microbiologic indicator for MTB infection in persons with LTBI. Further research is warranted to assess the effect of LTBI status on the performance of MTB-cfDNA detection to identify PTB in those suspected of having it.

Regarding factors correlated with MTB-cfDNA detectability in patients with PTB, Ushio et al. observed that male sex, bilateral lung lesions, and coexisting extrapulmonary lesion(s) were correlated with high concentrations of MTB-cfDNA [9]. Furthermore, we observed that MTB-specific IFN-γ response was associated with MTB-cfDNA positivity in patients with PTB. Although the mechanism is unknown, one possible explanation is that the antigen-presenting cells in MTB-cfDNA-negative patients are less likely to induce an MTB-specific adaptive immune response and release MTB-cfDNA into circulation than in MTB-cfDNA-positive patients. This is supported by a report stating that dysfunction in antigen-presenting cells was more prominent in patients with PTB and with reduced response to a purified protein derivative (PPD-anergy) than in those who were PPD-reactive [19]. Whether high MTB-cfDNA concentration in patients with PTB is associated with immune-specific phenotypes and treatment response warrants a more detailed investigation.

This study had some limitations. First, the sample size was relatively small. Second, although our protocols for cfDNA extraction and PCR were similar to those of Ushio et al. [9], the extraction kit we used was not specific for cfDNA; therefore, MTB-cfDNA detectability may have been underestimated [20]. Additional experiments optimizing cfDNA extraction and testing PCR by using different primer and probe sets for MTB-cfDNA detection are warranted [11, 21]. Third, although we demonstrated the presence of plasma MTB-cfDNA in two persons with LTBI as microbiologic evidence of MTB infection, the detection rate remained too low for clinical applications for LTBI management. Additional large-scale cohort studies using more sensitive methods to assess the value of detecting blood MTB-specific DNA in contact investigation and LTBI therapy monitoring are warranted [22].

In conclusion, circulating MTB-cfDNA was detectable in half of the patients with PTB. By combining the MTB-cfDNA and MLR results, sensitivity increased to nearly 80%. MTB-cfDNA positivity was associated with a higher MTB-specific IFN-γ response, the level of which decreased after treatment. By detecting MTB-cfDNA, this study also provided the first microbiologic evidence of MTB infection in persons with a history of TB contact and with LTBI. The adjuvant roles of MTB-cfDNA detection in the diagnosis of PTB and treatment response monitoring warrant more thorough investigations.

## Supporting information

S1 File (PDF)
