# Supplementary material for: Mycobacterium tuberculosis–derived circulating cell-free DNA in patients with pulmonary tuberculosis and persons with latent tuberculosis infection
Source: PLoS One. 2021 Jun 24;16(6):e0253879. doi: 10.1371/journal.pone.0253879 (PMC8224927; doi:10.1371/journal.pone.0253879)
Supplement: S1 File — (PDF) [file pone.0253879.s001.pdf]

Original article

Title: *Mycobacterium tuberculosis*–derived circulating cell–free DNA in patients with pulmonary tuberculosis and persons with latent tuberculosis infection

Authors: **Sheng-Wei Pan[a,b,c], Wei-Juin Su[a,b], Yu-Jiun Chan[c,d,e], Fan-Yi Chuang[a,b], Jia-Yih Feng[a,b], and Yuh-Min Chen[a,b]**

Institution:

[a]Department of Chest Medicine, Taipei Veterans General Hospital, Taipei, Taiwan

[b]School of Medicine, National Yang Ming Chiao Tung University, Taipei, Taiwan

[c]Institute of Public Health, National Yang Ming Chiao Tung University, Taipei, Taiwan

[d]Division of Infectious Diseases, Department of Medicine, Taipei Veterans General Hospital, Taipei, Taiwan

[e]Division of Microbiology, Department of Pathology and Laboratory Medicine, Taipei Veterans General Hospital, Taipei, Taiwan

Keywords: cell-free DNA; digital PCR; latent tuberculosis infection; pulmonary tuberculosis

Running Title: MTB-cfDNA in patients with TB and persons with LTBI

The corresponding authors:

Jia-Yih Feng, MD., PhD.

Department of Chest Medicine, Taipei Veterans General Hospital

No. 201, Sec. 2, Shih-Pai Rd., Taipei, Taiwan. E-mail: peterofeng@gmail.com

and

Sheng-Wei Pan, MD., PhD.

Department of Chest Medicine, Taipei Veterans General Hospital

No. 201, Sec. 2, Shih-Pai Rd., Taipei, Taiwan. E-mail: sanweipan@gmail.com

S1 File

Appendix 1. Results of *IS6110*-targeted quantitative polymerase chain reaction in plasma cell-free DNA from patients with pulmonary tuberculosis stratified by sputum smear grading.

Among 24 patients with pulmonary tuberculosis (PTB) in the present study, 11 were sputum-smear positive for acid-fast bacilli (4 with 4+ on the grading scale, 5 with 3+, 2 with 2+, and none with 1+). Table S1 presents the results of quantitative polymerase chain reaction (qPCR) targeting the *Mycobacterium tuberculosis* (MTB)-specific *IS6110* gene fragment in plasma cell-free DNA (cfDNA) in PTB patients stratified by sputum smear grading. The median cycle threshold (Ct) value was 37.24 in patients with 4+ smear-positivity, 37.92 in those with 2-3+, and 38.58 in those with smear-negativity. However, no significant difference in the Ct values was observed between the three groups (p = 0.958 in the analysis of variance). Additional large-scale studies to assess the relationship between the quantification of bacilli in sputum in patients with PTB and qPCR results are warranted.

Table S1. Results of qPCR targeting MTB-derived *IS6110* in the plasma cfDNA of patients with PTB stratified by sputum smear grading (n = 24).

Sputum smear grading	Cycle threshold value, Median (interquartile range)
Smear-positive, 4+ in grade (n=4)	37.24 (36.32-42.93)
Smear-positive, 2-3+ grade (n=7)	37.92 (33.57-45.00)
Smear-negative (n=13)	38.58 (33.98-45.00)
